# Comparison of the Effect of Spinal Anesthesia Applied in Elective Cesarean Cases on Frontal QRS Angle in Anemic and Non-Anemic Patients

**DOI:** 10.3390/jcm14217827

**Published:** 2025-11-04

**Authors:** Ahmet Kaya, Mahmut Alp Karahan, Mehmet Tercan, Alev Esercan, Melike Bostanci Erkmen, Omer Faruk Cicek

**Affiliations:** 1 Department of Anesthesiology and Reanimation, Mehmet Akif Inan Training and Research Hospital, University of Health Science, 63040 Sanliurfa, Turkey; mahmutalp_k@yahoo.com; 2 Department of Anesthesiology and Reanimation, Antalya Training and Research Hospital, University of Health Sciences, 07100 Antalya, Turkey; drmtercan@gmail.com; 3 Department of Obstetrics and Gynaecology, Kanuni Training and Research Hospital, University of Health Science, 61250 Trabzon, Turkey; alevesercan@gmail.com; 4 Department of Anesthesiology and Reanimation, Sanliurfa Training and Research Hospital, 63250 Sanliurfa, Turkey; melikeb63@hotmail.com; 5 Department of Cardiology, Mehmet Akif Inan Training and Research Hospital, University of Health Science, 63040 Sanliurfa, Turkey; omerfarcic@hotmail.com

**Keywords:** pregnancy, anemia, electrocardiography, QRS duration, frontal QRS-T angle, obstetric anesthesia, maternal cardiac assessment

## Abstract

**Background/Objectives**: Pregnancy is associated with profound physiological alterations that, together with anemia and spinal anesthesia, may influence myocardial repolarization. The frontal QRS-T [f(QRS-T)] angle has emerged as a reliable electrocardiographic parameter for evaluating repolarization heterogeneity. **Materials and Methods**: This observational prospective study included 100 term pregnant women [18–45 years, American Society of Anaesthesiologists (ASA) II] undergoing elective cesarean delivery under spinal anesthesia at Sanliurfa Training and Research Hospital between May and August 2025. Participants were divided into two groups: anemic (Hb < 10.5 g/dL, *n* = 50) and non-anemic (Hb ≥ 10.5 g/dL, *n* = 50). Standard monitoring and 12-lead ECGs were performed preoperatively and postoperatively. The f(QRS-T) angle was calculated as the absolute difference between QRS and T axes; values > 180° were adjusted by subtracting from 360°. **Results**: Demographic variables were comparable between groups. No significant differences were observed in mean arterial pressure or heart rate. Preoperative QTc and f(QRS-T) angle values did not differ significantly. However, postoperative QTc was prolonged in the anemic group compared with non-anemic women (426.3 ± 19.2 ms vs. 417.2 ± 20.7 ms, *p* = 0.026). Likewise, the postoperative f(QRS-T) angle was significantly higher in anemic patients (29.5 [16.0–45.3] vs. 20.5 [9.8–34.5], *p* = 0.017). Within-group analysis revealed significant postoperative increases in both QTc (*p* < 0.001) and f(QRS-T) angle (*p* < 0.001) in the anemic group, but not in controls. Hemoglobin levels correlated negatively with postoperative QTc (r = −0.267, *p* = 0.008) and f(QRS-T) angle (r = −0.264, *p* = 0.008). **Conclusions**: In anemic patients undergoing cesarean delivery under spinal anesthesia, the postoperative QTc interval and f(QRS-T) angle increased significantly compared with both baseline values and non-anemic counterparts. Assessment of the f(QRS-T) angle, a simple and inexpensive ECG-derived parameter, may aid in perioperative risk stratification and enhance patient safety.

## 1. Introduction

Pregnancy is associated with extensive physiological alterations that significantly influence both the mother and the fetus. Maternal blood volume physiologically increases by approximately 30–50%, whereas erythrocyte volume rises by 17–30%. The initial increase in circulating volume is observed during the first trimester, reaches its peak in the second trimester, and subsequently declines. Since the increase in plasma volume exceeds that of the erythrocyte volume, hemodilution occurs, a phenomenon termed physiological iron-deficiency anemia [1].

Anemia in pregnancy is defined as a reduced hemoglobin concentration, with a level below 11.0 g/dL in the first trimester and less than 10.5–11.0 g/dL in the second or third trimester, depending on the reference guideline. It represents the most common hematologic disorder during pregnancy [2], has been linked to poor cardiovascular outcomes and is considered a strong predictor of cardiac mortality. The aforementioned factors have been demonstrated to induce myocardial structural and electrophysiological changes, including hypertrophy at left ventricle, increased heart rate, elevated filling pressures, and ischemia. These alterations are the result of an imbalance between oxygen supply and demand [3].

Pregnancy itself imposes considerable stress on the cardiovascular system by increasing cardiac output, heart rate, circulating volume, and venous pressure in the lower extremities, while reducing systemic vascular resistance, pulmonary vascular resistance, and blood pressure. In addition, it affects the cardiac conduction system, predisposing patients to arrhythmias. Atrial arrhythmias are the most common, and ventricular tachyarrhythmias, though rare, may occur during pregnancy and can be life-threatening [4].

Cesarean section, a frequently performed surgical procedure in pregnant women, may be necessary for anemic patients as well. Various anesthetic techniques—including general, spinal, and epidural anesthesia—are utilized in such cases. Spinal anesthesia is particularly preferred due to its rapid onset, relative ease of administration, and high success rate. However, in addition to these benefits, it may cause cardiovascular side effects such as sympathetic block-related vasodilation, reductions in right atrial pressure, and reflex bradycardia (Bainbridge reflex), depending on the block level [5].

**Figure 1:**
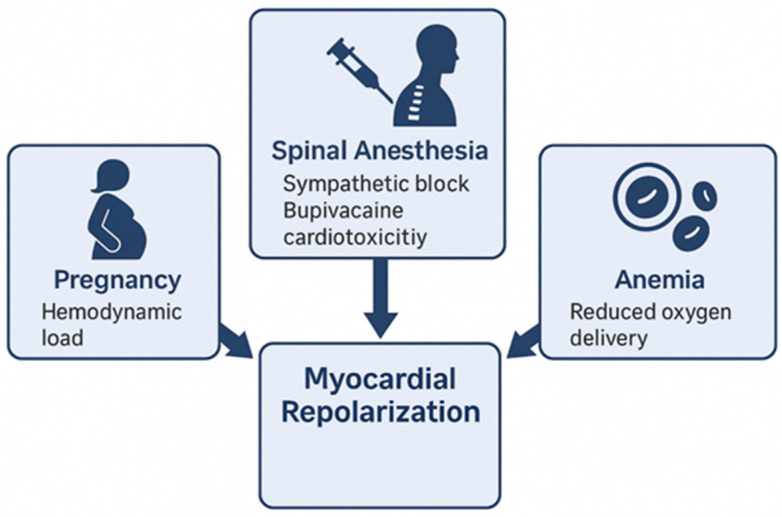
Factors Influencing Myocardial Repolarization in Anemic Pregnant Women Undergoing Spinal Anesthesia.

The coexistence of anemia, pregnancy-related cardiovascular changes, the spinal anesthesia technique, and bupivacaine’s cardiotoxic effects may influence repolarization and depolarization ofmyocardium by altering the generation and propagation of electrical impulses (Figure 1). Therefore, this study aimed to evaluate the influence of spinal anesthesia on the frontal QRS-T angle in anemic patients undergoing elective cesarean section.

## 2. Materials and Methods

### 2.1. Study Design

This observational prospective study was conducted between 15 May 2025 and 29 August 2025 in the operating theaters of Sanliurfa Training and Research Hospital. Ethical approval was obtained from the Clinical Ethics Committee of Harran University (date: 15 April 2024; number: HRU/24.04.04).

### 2.2. Patient Selection

The sample size was calculated based on the results of the first 15 patients included in the study. It was determined that a minimum of 98 patients were required for the study, based on the observed differences and under the assumption of a one-tailed α value of 0.05 (sensitivity: 95%) and β value of 0.05 (study power: 95%, effect size: 0.67). This determination was made using the G-Power 3.1.9.7 programfor power analysis. To account for potential dropouts, the sample size was increased by 10%, resulting in 54 patients per group. The study comprised a total of 108 patients; of these, 3 were excluded because they underwent general anesthesia and 5 were excluded as they did not meet the criteria.

A total of 100 term pregnant women, aged 18–45 years, classified as American Society of Anaesthesiologists (ASA) II and scheduled for elective cesarean section were recruited. The notion of elective cesarean section has been employed in the context of non-urgent cases. All participants were fully informed about comprehensive verbal and written information regarding the study prior to their enrollment, and written informed consent was duly obtained from each participant. Exclusion criteria included systemic diseases (e.g., Diabetes, Chronic Obstructive Pulmonary Disease (COPD), Hypertension), Body Mass Index (BMI) > 30, multiple gestation, pre-eclampsia/eclampsia, preterm or post-term pregnancy, fetal abnormalities, Rh incompatibility, hypersensitivity to local anesthetics, and any contraindication to regional anesthesia. Patients with arrhythmias or electrolyte imbalances were also excluded. Furthermore, individuals who required general anesthesia or any medication likely to affect blood pressure prior to delivery were removed from the analysis.

### 2.3. Anesthesia Management

A total of 100 patients were enrolled and evenly assigned to two groups, Group A (anemic, preoperative hemoglobin < 10.5 g/dL) and Group AO (non-anemic, hemoglobin ≥ 10.5 g/dL), with 50 patients allocated to each group. Preoperative demographic and clinical variables were recorded based on medical chart review and direct interviews. Intraoperatively, patients were positioned in the supine position, and standard monitoring was implemented, including electrocardiography (ECG), heart rate (HR), non-invasive arterial pressure, and peripheral oxygen saturation. Baseline values were recorded before spinal anesthesia.

All patients were placed in a sitting position to facilitate administration of spinal anesthesia. After sterile preparation of the skin, the L3–L4 intervertebral space was identified. A 27G pencil-point spinal needle (Egemen, Istanbul, Turkey) was inserted into the subarachnoid space, and following free cerebrospinal fluid flow, 1.7 mL (8.5 mg) of 0.5% hyperbaric bupivacaine (Marcaine 0.5%, AstraZeneca, UK) was injected. Patients were then positioned supine and received oxygen at 3 L/min via nasal cannula.

After administration, the anesthesiologist assessed autonomic block with an alcohol swab, sensory block by pinprick testing, and motor block according to the Bromage scale (grade 3). Surgical intervention commenced once an adequate sensory block extending to the T5–T6 level was established. Only patients with a successful spinal block were included in the study.

### 2.4. Electrocardiography

Standard 12-lead ECGs were performed preoperatively and postoperatively in the supine position using the Cardiofax M Model ECG-1250 system (Nihon Kohden Corporation, Tokyo, Japan), with settings of 25 mm/s paper at speed and a 10 mm/mV calibration.

Automated ECG reports provided the T and frontal QRS axes, which were subsequently verified. The angle of f(QRS-T) was determined by calculating the absolute difference between the axes of QRS and T (QRS-T angle = QRS axis – T axis). When the resulting value exceeded 180°, it was recalculated by subtracting the obtained measurement from 360° [6]. All ECG data were independently reviewed by cardiologists.

### 2.5. Statistical Analysis

All statistical data were analyzed with Statistical Package for the Social Sciences (SPSS) software, version 22.0. The Kolmogorov–Smirnov test was employed to assess the normality of continuous variables. Continuous variables with normal distribution were summarized as the mean ± standard deviation, whereas those not normally distributed were described as the median (Q1–Q3). Between-group comparisons were carried out using the independent-samples *t*-test for parametric variables and the Mann–Whitney U test for nonparametric variables. Dichotomous variables were expressed as percentages and frequencies [*n* (%)] and compared using the chi-square test. Paired pre- and postoperative measurements within groups were analyzed using the pairedsamples *t*-test or the Wilcoxon signed-rank test, as appropriate. For repeated measures involving more than two time points, a repeated-measures ANOVA was applied. Correlations between variables were assessed using Pearson or Spearman correlation coefficients. A two-tailed *p*-value < 0.05 was considered statistically significant.

## 3. Results

A total of 100 patients undergoing cesarean delivery were included in the study: 50 with anemia and 50 without anemia. The mean hemoglobin levels of the anemic and non-anemic groups were 9.60 ± 0.54 g/dL and 11.59 ± 0.94 g/dL, respectively. The investigation revealed no statistically substantial differences between the two groups with respect to age (*p* = 0.407), weight (*p* = 0.474), height (*p* = 0.376), or gravida (*p* = 0.300) (Table 1).

The perioperative changes in mean arterial pressure (MAP) are presented in Table 2. There were no significant differences between the anemic and non-anemic groups at the same time points. Similarly, heart rate (HR) variations are summarized in Table 3. Although baseline (*p* = 0.081) and postoperative (*p* = 0.073) HR tended to be higher in the anemic group, no statistically significant differences were present.

QTc intervals and frontal QRS-T angle measurements are presented in Table 4. Preoperative QTc intervals did not differ significantly between groups (*p* = 0.115). However, postoperative QTc intervals were significantly prolonged in anemic women compared to their non-anemic counterparts (426.33 ± 19.23 ms vs. 417.24 ± 20.73 ms, *p* = 0.026). Similarly, the preoperative frontal QRS-T angle showed no significant difference (*p* = 0.075), while the postoperative f(QRS-T) angle was markedly higher in the anemic group compared with the non-anemic group (29.5 [16.0–45.3] vs. 20.5 [9.8–34.5], *p* = 0.017).

Within-group comparisons demonstrated that in the anemic group, both intervals of the QTc (*p* < 0.001) and f(QRS-T) angle (*p* < 0.001) increased significantly postoperatively compared with preoperative values, whereas no significant changes were observed in the non-anemic group.

**Figure 2:**
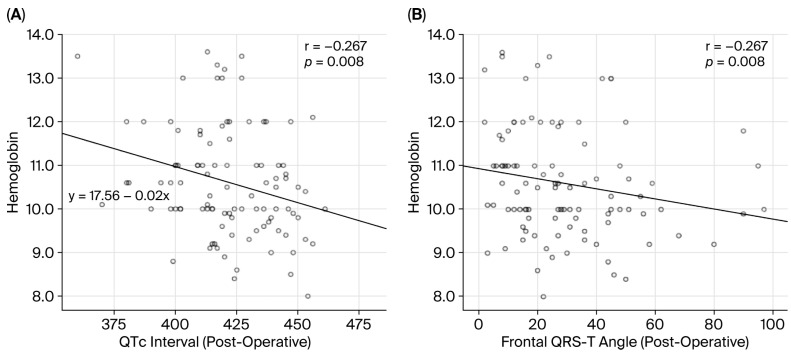
Correlation of hemoglobin count with (**A**) postoperative QTc interval, and (**B**) postoperative frontal QRS-T angle.

Correlation analysis revealed that hemoglobin concentration was correlated negatively with postoperative interval of QTc (r = −0.267, *p* = 0.008) and postoperative angle of f(QRS-T) (r = −0.264, *p* = 0.008) (Figure 2A,B). No significant associations were found between hemoglobin levels and preoperative QTc interval or frontal QRS-T angle.

## 4. Discussion

In this study, we investigated term pregnancies undergoing cesarean delivery with spinal anesthesia and found that postoperative angle of f(QRS-T) was significantly higher in the anemic group compared to the non-anemic group; within the anemic group, the postoperative angle of f(QRS-T) was significantly greater than the preoperative measurements, and QTc interval changes paralleled the alterations observed in the angle of f(QRS-T).

Electrocardiographic markers such as the interval of QT (QT), dispersion of QT (QTd), corrected QT interval (QTc), transmural dispersion of repolarization (TDR), and Tp-e/QT or Tp-e/QTc ratios are widely employed as indicators of ventricular arrhythmias [7]. However, limitations including a lack of standardization and uncertain predictive value reduce their clinical applicability. As a result, novel indices have been proposed. Among the available parameters, the angle of f(QRS-T)—characterized as the angular difference between ventricular depolarization and repolarization vectors—has been increasingly recognized as a promising measure of ventricular repolarization heterogeneity. Previous studies have demonstrated its prognostic value in various clinical populations. While the spatial QRS-T angle requires specialized software, the f(QRS-T) angle can be easily derived from routine ECG recordings, as axes of QRS and T are automatically provided by most modern ECG devices [8].

Individually, anemia, pregnancy, and spinal anesthesia each have the potential to influence myocardial repolarization. These conditions often coexist in anemic patients undergoing cesarean delivery with spinal anesthesia. To the best of our knowledge, the cumulative impact of these factors on repolarization at myocardium has not been systematically evaluated in previous studies. Furthermore, available studies on the relationship between anemia, pregnancy, cesarean section, and spinal anesthesia with the frontal QRS-T angle remain limited.

It is well established that pregnancy is linked to a higher prevalence of arrhythmias. In particular, benign arrhythmias, including premature atrial and ventricular contractions, are observed more commonly in pregnant women than in the non-pregnant period. A prior history of arrhythmia constitutes a significant risk factor for recurrence during gestation. Even in structurally normal hearts, de novo ventricular tachycardia may occur. The mechanisms underlying these proarrhythmic effects include cardiovascular, autonomic, and hormonal alterations, such as elevated plasma catecholamine levels, relaxin’s chronotropic effects, atrial stretch, increasing of end-diastolic volume from intravascular expansion, and both emotional and hormonal changes. During pregnancy, there is a gradual increase in heart rate, resulting in a 10–25% increase compared to pre-pregnancy values. Cardiac output increases during pregnancy, with a 45% rise occurring in cardiac output at the onset of the second trimester. Estrogen and relaxin have been demonstrated to stimulate the production of nitric oxide, thereby increasing peripheral arterial compliance and reducing resistance of vascularity [9].

During normal pregnancy, plasma volume increases by 40–60%, while red blood cell mass increases by only 20–50%, leading to physiological anemia with a hematocrit of 30–32%. In anemic pregnancies, the reduced oxygen-carrying capacity further increases cardiovascular strain, potentially predisposing to arrhythmias. Severe anemia may result in circulatory decompensation, elevated cardiac output, increased bleeding risk, impaired tolerance to blood loss, and in some cases, circulatory collapse or death [10]. Severe anemia can precipitate circulatory decompensation with compensatory increases in cardiac output, elevated bleeding risk, and impaired capacity to withstand blood loss, which in turn may culminate in circulatory shock and mortality [11]. Anemia not only induces structural and hemodynamic changes, such as left ventricular hypertrophy and increased cardiac output, but may also cause electrical disturbances, including depolarization and repolarization abnormalities. Hemoglobin concentration has been reported as an independent determinant of the angle of f(QRS-T) [3]. Consistent with these findings, our correlation analysis revealed that hemoglobin level was correlated negatively with the postoperative interval of QTc (r = −0.267, *p* = 0.008) and f(QRS-T) angle (r = −0.264, *p* = 0.008).

Bupivacaine, a commonly used local anesthetic in spinal anesthesia, is known to exhibit cardiotoxic effects, even at relatively low plasma concentrations. Reported complications include supraventricular tachycardia, atrioventricular block, premature ventricular contractions, QRS abnormalities, and fatal ventricular fibrillation [12]. Several studies have explored the impact of spinal anesthesia on QT intervals during cesarean delivery. For instance, spinal anesthesia has been shown to prolong QTc intervals more markedly in diabetic women than in their non-diabetic counterparts [13]. Other studies have demonstrated that QT dispersion is significantly increased in patients at ≥39 weeks of gestation undergoing spinal anesthesia [14]. Furthermore, women with severe pre-eclampsia exhibited more pronounced QT interval prolongation compared to normotensive patients [15]. Comparative studies of local anesthetics have suggested that levobupivacaine provides superior hemodynamic stability, whereas bupivacaine exerts a greater impact on QTc prolongation and QT dispersion, indicating that levobupivacaine may be a safer alternative, particularly in patients with cardiovascular risk [16,17].

To date, few studies have examined the angle of f(QRS-T) in patients. One investigation reported increased angle of f(QRS-T) in patients with pre-eclampsia compared to healthy pregnancies, indicating its possible utility as an independent predictor of pre-eclampsia onset [18]. In addition, two recent studies evaluated f(QRS-T) angle in cesarean deliveries performed under spinal anesthesia. The initial study demonstrated that in post-term pregnancies, both prolonged duration of QRS and elevated f(QRS-T) angle may serve as markers of electrophysiological changes arising from advanced gestational age [19]. The second study showed that body mass index (BMI) ≥ 30 was linked to a statistically significant increase in the angle of f(QRS-T) observed in pregnant women receiving spinal anesthesia for cesarean section, whereas no significant changes were observed in women with BMI < 30 [20].

Collectively, these findings indicate that anemia, pregnancy, and spinal anesthesia may each increase the frontal QRS-T angle, thereby influencing myocardial repolarization. However, no prior study has specifically focused on anemic women as a subgroup. To our knowledge, this study is the first to comprehensively examine the interaction of anemia, pregnancy, and spinal anesthesia in relation to the angle of f(QRS-T). Our findings revealed that in term patients undergoing cesarean section with spinal anesthesia, the postoperative angle of frontal QRS-T and interval of QTc were significantly increased in the group of anemic patients compared both to the preoperative values and those of thenon-anemic controls.

### Limitations

This study has several limitations. First, the effects of benign or malignant ventricular arrhythmias were not evaluated. Continuous Holter monitoring could have revealed a potential association between the angle of f(QRS-T) and ventricular arrhythmias.

Second, patients were not reassessed after correction of anemia. Determining whether the angle of f(QRS-T) narrows following treatment of anemia would be of clinical interest.

Third, serial ECG recordings were not obtained, and long-term follow-up data were lacking. Therefore, the impact of the angle of f(QRS-T) on long-term outcomes remains unclear. Larger randomized clinical trials with extended follow-up are necessary to validate these findings and to establish clinically applicable threshold values for the f(QRS-T) angle.

Despite the fact that an increased angle of f(QRS-T) is considered a marker of arrhythmogenic risk, no cases of malignant arrhythmia were identified among the study participants.

## 5. Conclusions

To conclude, the angle of f(QRS-T) constitutes a straightforward, economical, and readily measurable index available from conventional surface ECG. Our findings revealed that in anemic patients who received spinal anesthesia for cesarean section, postoperative prolongation of the frontal QRS-T angle was significant when compared both to their baseline values and to those observed in non-anemic patients.

The assessment of the angle of f(QRS-T) in anesthetic practice may provide a practical tool to identify patients at increased risk of arrhythmias. Incorporating this parameter into perioperative evaluation could contribute to improved risk stratification and patient safety.

## Figures and Tables

**Table 1:** Baseline clinical characteristics of the pregnant with and without anemia. (kg: kilogram, cm: centimeter, n: number of patients, g/dL: gram/deciliter).

Variables	Anemic Group (n = 50)	Non-Anemic Group (n = 50)	*p*
Age, years, mean ± sd	28.50 ± 4.63	27.78 ± 4.00	0.407 ^a^
Weight, kg, mean ± sd	72.62 ± 10.66	71.10 ± 10.48	0.474 ^a^
Height, cm, mean ± sd	161.22 ± 5.43	160.26 ± 5.38	0.376 ^a^
Gravida, n, median (min-max)	4 (3–5)	4 (3–6)	0.300 ^b^
Hemoglobin, g/dL, mean ± sd	9.60 ± 0.54	11.59 ± 0.94	<0.001 ^a^

^a^ Student’s *t*-test, ^b^ Mann–Whitney U-test.

**Table 2:** Mean arterial pressure (MAP) changes in pregnant women with and without anemia.

	**Anemic Group** **(n = 50)**	**Non-Anemic Group** **(n = 50)**	***p***
MAP (mmHg)			
preoperative	89.50 ± 15.48	87.20 ± 14.52	0.479 ^a^
1st minute	84.90 ± 14.19	82.04 ± 14.15	0.332 ^a^
10th minute	73.92 ± 14.43	72.06 ± 11.97	0.560 ^a^
postoperative	77.44 ± 13.95	70.48 ± 20.73	0.055 ^a^
	*p* < 0.001 ^c^	*p* < 0.001 ^c^	

^a^ Student’s *t*-test, ^c^ repeated measures ANOVA.

**Table 3:** Heart rate (HR) changes in pregnant women with and without anemia. (bpm: beats per minute).

	Anemic Group (n = 50)	Non-Anemic Group (n = 50)	*p*
HR (bpm)			
preoperative	102.28 ± 10.42	96.98 ± 18.44	0.081 ^a^
1st minute	105.81 ± 13.87	103.94 ± 14.62	0.635 ^a^
10th minute	104.85 ± 20.01	106.51 ± 10.07	0.532 ^a^
postoperative	94.50 ± 17.94	88.22 ± 16.39	0.073 ^a^
	*p* < 0.001 ^c^	*p* < 0.001 ^c^	

^a^ Student’s *t*-test, ^c^ repeated measures ANOVA.

**Table 4:** Corrected QT (QTc) interval and frontal QRS-T angle changes in pregnant women with and without anemia.

	Anemic Group (n = 50)	Non-Anemic Group (n = 50)	*p*
**QTc, ms** Preoperative Postoperative	411.55 ± 13.58 426.33 ± 19.23	416.56 ± 19.78 417.24 ± 20.73	0.115 ^a^ 0.026 ^a^
	*p* < 0.001 ^d^	*p* = 0.852 ^d^	
**Frontal QRS-T angle (°)** Preoperative Postoperative	23.0 (10.8–33.3) 29.5 (16.0–45.3)	13.0 (6.0–29.3) 20.5 (9.8–34.5)	0.075 ^b^ 0.017 ^b^
	*p* < 0.001 ^e^	*p* = 0.094 ^e^	

^a^ Student’s *t*-test, ^b^ Mann–Whitney U-test, ^d^ Paired samples *t*-test, ^e^ Wilcoxon test.

## Data Availability

The original contributions presented in this study are included in the article; further inquiries can be directed to the corresponding author.

## Abbreviations

The following abbreviations are used in this manuscript:

ECG	electrocardiography
f(QRS-T)	frontal QRS-T angle
ASA	American Society of Anesthesiology
Hb	Hemoglobin
QTc	corrected QT
BMI	Body Mass Index
HR	heart rate
SPSS	Statistical Package for the Social Sciences
MAP	Mean arterial pressure
QTd	dispersion of QT
